# Distal 22q11.2 Microduplication: Case Report and Review of the Literature

**DOI:** 10.1177/2329048X17737651

**Published:** 2017-11-01

**Authors:** Elana Pinchefsky, Laurence Laneuville, Myriam Srour

**Affiliations:** 1 Division of Pediatric Neurology, Department of Pediatrics, Montreal Children’s Hospital, McGill University Health Centre (MUHC), Montreal, Québec, Canada; 2 Department of Medicine, McGill University, Montreal, Québec, Canada

**Keywords:** developmental delay, genetics, intellectual disability, neurodevelopment, pediatric

## Abstract

Distal chromosome 22q11.2 microduplications are associated with a wide range of phenotypes and unclear pathogenicity. The authors report on a 3-year-old girl with global developmental delay harboring a de novo 1.24 Mb distal chromosome 22q11.2 microduplication and a paternally inherited 0.25 Mb chromosome 4p14 microduplication. The authors review clinical features of 30 reported cases of distal 22q11.2 duplications. Common features include developmental delay (93%), neuropsychiatric features (26%), and nonspecific facial dysmorphisms (74%). In 70% of cases, the distal 22q11.2 duplications were inherited, and the majority of the carrier parents were phenotypically normal. Furthermore, 30% of probands carried an additional copy number variant. Review of the phenotype in individuals carrying microduplications involving similar low copy repeats (LCR) failed to establish any clear genotype–phenotype correlations. Distal 22q11.2 duplications represent a major challenge for genetic counseling and prediction of clinical consequences. Our report suggests a pathogenic role of distal 22q11.2 duplications and supports a “multiple hit” hypothesis underlying its variable expressivity and phenotypic severity.

Chromosome 22q11.2 deletions, which occur in up to 1:4000 to 6000 live births, are among the most frequently reported chromosomal rearrangements.^1^ This region is highly susceptible to microdeletions and microduplications due to the presence of LCR which mediate nonallelic homologous recombination and predispose the region to chromosomal rearrangements.^2-5^ The 22q11.2 regions contains 8 LCR designated as LCR A-H.^5^ The pathogenicity of deletions of chromosome 22q11.2 is well recognized. Both the recurrent 3 Mb (LCR A-D) and 1.5 Mb (LCR A-B) proximal deletions result in DiGeorge/velocardiofacial syndrome.^6^ The distal 22q11.2 microdeletion syndrome (LCR D-H) is characterized by variable clinical features and includes global developmental delay, intellectual disability, mild dysmorphic features, pre- and/or postnatal growth restriction, cardiovascular defects, and a high incidence of malignant rhabdoid tumors in infancy and early childhood. The deletions are de novo in the majority of cases but are occasionally inherited from a parent with a mild or normal phenotype.^7,8^

To date, over 40 patients with distal 22q11.2 microduplications (LCR D-H) have been reported. The associated phenotype has been variable and includes developmental/intellectual delay, speech or language disturbances, behavior problems, hyperactivity, epilepsy, hypotonia, dysmorphic facial features, and congenital heart defects and rarely hearing loss, cleft lip, and urogenital abnormalities.^2,7,9-15^ However, many familial cases and carrier parents with normal phenotypes have been reported.^2,15^ The varied phenotype reported with distal 22q11.2 duplications constitutes a major challenge to clinicians with regard to genetic counseling and prediction of clinical consequences.

Here, the authors report on the clinical phenotype and cytogenetic studies of a 3-year-old girl with global developmental delay, autistic features, and sensorineural hearing loss, who harbors a de novo distal chromosome 22q11.2 duplication. The authors also review the literature pertaining to the pathogenicity of distal 22q11.2 duplications and compare the clinical features of 28 previously published cases.

## Clinical Report

The patient was first evaluated in the neurology clinic at age 2.5 years for global developmental delay. She was born to healthy nonconsanguineous parents of Moroccan origin and has 2 healthy developmentally normal siblings. She was the product of an unremarkable pregnancy. Delivery was spontaneous, vaginal, and uncomplicated. Birth weight was 3955 g (98th percentile), height was 52 cm (90th percentile), and head circumference was 36 cm (97th percentile). She developed febrile seizures at age 9 months, then afebrile generalized seizures well controlled on clobazam. Bilateral mild-to-moderate sensorineural hearing loss was noted at age 23 months. Her development was globally delayed. She walked at the appropriate age of 12 months but could not go down stairs, jump, or kick a ball at age 39 months. Between the ages of 12 and 20 months, she acquired approximately 10 words, which she subsequently lost. She currently has no word output but can understand simple instructions. She feeds herself with her hands but does not scribble or dress herself. She has inconsistent eye contact and does not point to her wants. She prefers to play on her own but will interact with others if prompted. She does not engage in pretend play. She is hyperactive and has difficult behavior and occasional tantrums. Her parents have noted bruxism. She started clonidine with improvement in attention deficit and behavior. On formal developmental assessment at age 22 months, she had a significant delay in gross and fine motor development. She performed at the first percentile for age using the Peabody Developmental Motor Scales. She also had a severe receptive and expressive language delay, with some signs of language regression noted at age 30 months. On her most recent examination at age 3 years, her weight was 18.3 kg (90th percentile), height was 87 cm (2nd percentile), and head circumference was 48.5 cm (25th percentile). She had mild dysmorphic features with a thin upper lip, slightly long and well-defined philtrum, mild retromicrognathia, a bulbous nose, and arched eyebrows. Her tone was increased in her lower extremities with tight heel cords. She walked on her toes. Deep tendon reflexes were brisk. She wore articulated ankle–foot orthoses.

Investigations which included blood gas, complete blood count, liver function tests, lactate, ammonia, plasma amino acids, urine organic acids, acylcarnitine profile, creatine and guanidinoacetate, oligosaccharides and mucopolysaccharides, purines and pyrimidines, electromyogram and nerve conduction studies, methylation studies for Angelman, *GJB2* (Connexin-26) and *UBE3A* sequencing, and *FMR1* molecular analysis were normal. At age 3 years, her brain magnetic resonance imaging demonstrated delayed myelination bilaterally, with normal resonance spectroscopy. Computed tomography of the temporal bones was normal. Electroencephalogram showed a mild-to-moderate disturbance of cerebral activity in the form of paroxysmal activity, which was not clearly epileptiform.

Microarray-based comparative genomic hybridization (aCGH) analysis was performed using Nimblegen CGX-12 (Roche, Wisconsin, USA) containing approximately 135 000 oligonucleotides that are distributed in all regions implicated in cytogenetic anomalies including subtelomeric regions, pericentromeric regions, and over 200 genetic syndromes. All procedures were carried out according to the manufacturer’s protocol. The aCGH analysis revealed 2 copy number variations: (1) a 0.251 Mb duplication on chromosome 4p14 (chr 4: 38 547 661-38 798 575) and (2) a 1.241 Mb duplication on chromosome 22q11.23 (chr22: 22 080 929-23 321 669, hg18 assembly). The chromosome 4p14 duplication was found to be inherited from her phenotypically normal father and was felt to be of no direct clinical significance. The 22q11.23 duplication was confirmed by fluorescent in situ hybridization analysis and was found to be absent in both parents. It encompassed the following genes: *IGLL1, C22orf43, GUSBP11, RGL4, ZNF70, VPREB3, C22orf15, CHCHD10, MMP11, SMARCB1, DERL3, SLC2A11, LOC284889, MIF, GSTT2B, GSTT2, DDTL, DDT, GSTTP1, LOC391322, GSTT1, GSTTP2, CABIN1, SUSD2, GGT5, POM121L9P, SPECC1 L, ADORA2A, C22orf45, UPB1, C22orf13, SNRPD3, GGT1, and C22orf36.* Because the 22q11.23 duplication was de novo in our patient, and copy number variations in this region have previously been associated with neurodevelopmental disabilities,^2,7,9-15^ the chromosome 22q11.23 duplication was felt to contribute to the patient’s phenotype, though the presence of direct causality is still not clear.

## Discussion and Literature Review

The authors identified a 1.24 Mb de novo distal microduplication on chromosome 22q11.23 and a 0.25 Mb paternally inherited duplication on chromosome 4p14 in a patient with moderate global developmental delay, attention deficit hyperactivity disorder, autistic features, febrile seizures, sensorineural hearing loss, and spasticity. Because the chromosome 4p14 duplication was inherited from a phenotypically normal parent and has not previously reported to be associated with neurodevelopmental abnormalities, it was felt to be of no clinical significance. The significance of the de novo 22q11.2 duplication as it pertains to our patient’s phenotype, however, is less certain.

**Figure 1.**
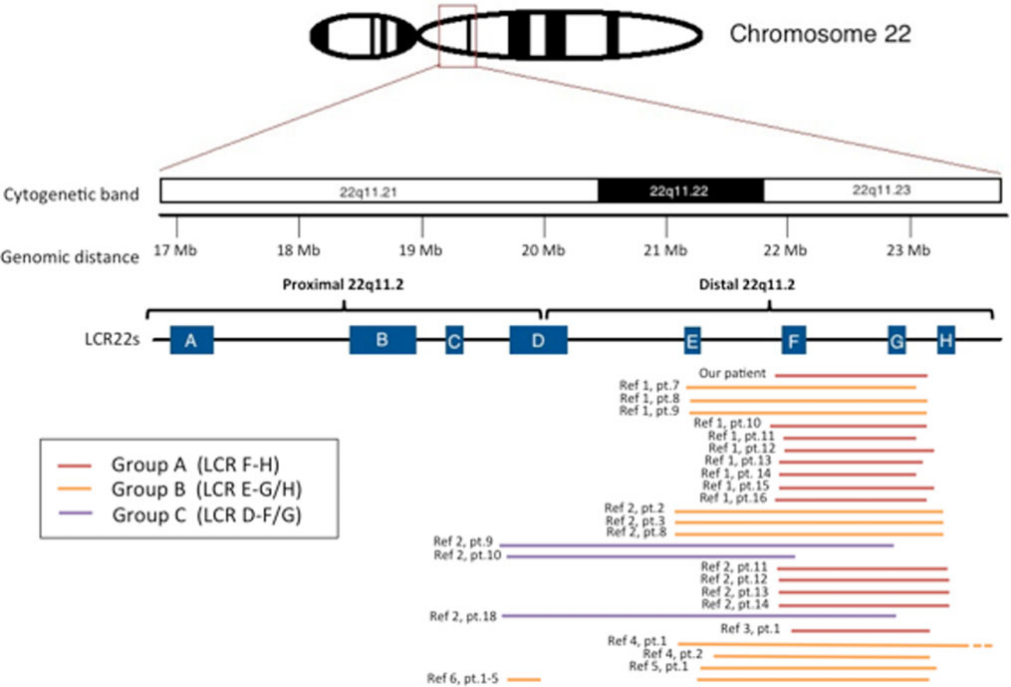
Schematic overview of overlapping distal 22q11.2 duplications from our patient and from previous literature.^2,7,9,10,12,15^

Although de novo copy number variations in children with global developmental delay are generally considered to be pathogenic, the significance of copy number variations in regions that are prone to rearrangements, such as chromosome 22q11.2, is less clear. The authors reviewed the phenotypic features of 30 previously reported patients with distal 22q11.2 microduplications overlapping with the one of our patients for whom clinical data were available (Figure 1, Table 1; Supplemental Table 1).^2,7,9,10,12,15^ The authors grouped together individuals with duplications involving the same LCR for comparison. The clinical features of 13 individuals with the duplications only involving the distal LCR F-H (including our patient) are summarized in Table 1. All patients had variable degrees of developmental delay: 15% (2/13) had isolated language delay and 85% (7/9) had global developmental delay ranging from mild to profound (information unavailable in 2 individuals). Epilepsy and neuropsychiatric features (including autistic traits) were noted in approximately one-third (2/7 and 3/8, respectively). No other individual in the group besides our patient was described as having spasticity or hearing loss. Duplications were inherited in 10 (77%) of 13 cases, of which 6 were of maternal origin and 4 of paternal origin. When the phenotype of the carrier parent was known, it was normal. Interestingly, 23% (3/13) of affected individuals had a second copy number variation.

**Table 1.** Summary of Clinical Features in Previously Reported Patients With Distal Chromosome 22q11.2 Microduplications Overlapping With Our Patient’s Phenotype.

	LCR F-H Microduplication	LCR E-G/H Microduplication	LCR D-F/G Microduplication	Total
Number of patients	13	14	3	30
Inheritance of CNV				
De novo	2	1	1	4
Inherited (M/P)	10 (6/4)	10 (4/6)	1 (1/-)	21 (11/10)
Unknown	1	3	1	5
Phenotype of carrier parent	Normal: 4; abnormal: 0; unknown: 6	Normal: 2; abnormal^a^: 6; unknown: 2	Unknown: 1	Normal: 6; abnormal^a^: 6; unknown: 9
Additional CNV	3/13 (23%)	6/14 (42%)^a^	0/3	9/30 (30%)^a^
Clinical features				
Developmental delay	13/13 (100%)	13/14 (93%)	2/3 (67%)	28/30 (93%)
GDD	8/10 (80%)	11/14 (79%)	1/3 (33%)	20/28 (71%)
Severity of GDD	Mild: 1; moderate: 2; severe: 1; profound: 1; unknown: 2	Mild: 2; moderate: 2; severe: 2; unknown: 5	Unknown: 1	Mild: 3; moderate: 4; severe: 3; profound: 1; unknown: 7
Language delay	9/9 (100%)	12/13 (92%)	1/3 (33%)	22/25 (88%)
Neuropsychiatric features	3/8 (38%)	2/12 (17%)	1/3 (33%)	6/23 (26%)
Seizures	2/7 (29%)	5/14 (36%)	0/3 (0%)	7/24 (29%)
Hypotonia	5/8 (63%)	2/8 (25%)	1/3 (33%)	8/19 (42%)
Facial dysmorphism	5/9 (56%)	12/14 (86%)	3/3 (100%)	20/27 (74%)
Macrocephaly	1/5 (20%)	3/11 (27%)	0/3 (0%)	4/19 (21%)
Microcephaly	1/5 (20%)	1/11 (9%)	1/3 (33%)	3/19 (16%)
Cleft lip	1/5 (20%)	0/11 (0%)	0/3 (0%)	1/19 (5%)
Cardiac abnormalities	0/7 (0%)	2/8 (25%)	1/3 (33%)	3/18 (17%)
Urogenital abnormalities	2/3 (67%)	1/6 (17%)	0/3 (0%)	3/12 (25%)
Skeletal defects	1/4 (25%)	3/5 (60%)	2/3 (67%)	6/12 (50%)

Abbreviations: CNV, copy number variant; GDD, global developmental delay; LCR, LCR; M, maternal; P, paternal.

^a^Note that 5 individuals from the same family from Descartes et al^15^ included, with all individuals carrying the same additional CNV (see text).

Review of the phenotypes of individuals carrying duplications involving LCR E-G/H (total = 14) or LCR D-F/G (total = 3) did not reveal any clear genotype–phenotype correlation. In particular, patients bearing larger duplications did not seem to have a more severe phenotype. Individuals bearing the duplications had variable development ranging from normal to severe delay, with variable presence of epilepsy (5/14 in LCR E-F duplication; 0/3 in D-F/G duplication) and neuropsychiatric features (2/12, 1/3).^2,7,9-15^ As with distal 22q11 duplications involving LCR F-H, many of the LCR E-G/H and D-F/G duplications were inherited from phenotypically normal parents. One notable exception is the multiplex family reported by Descartes et al^15^ harboring both a 22q11 LCR E-F duplication and a smaller 0.3 Mb LCR 22-D segregating in 5 individuals with developmental delay. One family member with the same 2 copy number variations was reported as having normal development at age 2 years.

Overall, the presence of distal 22q11 duplication in phenotypically normal parents and the reported variable-associated phenotype make it difficult to ascribe definite pathogenicity to these duplications.

Genomic deletions and duplications, including the distal chromosome 22q11.2 microduplication, are enriched in individuals with neurodevelopmental and neurobehavioral phenotypes.^16,17^ One to two percent of control individuals carry a variant greater than 1 Mb in length, compared to 7.8% to 20% of patients with idiopathic intellectual disability or global developmental delay.^16,18-24^ One possible explanation for the large phenotypic variability associated with copy number variations is the “multiple hit” model, in which a second insult is needed to produce a more severe clinical phenotype, via an additive or synergistic effect on neurodevelopmental pathways.^17,25-27^ The second hit could potentially be another copy number variation, a disruptive single base-pair mutation in a phenotypically related gene or even an environmental event.^17,25^ Ten percent of individuals with intellectual disability/global developmental delay who carry a primary copy number variant have at least 1 additional large variant. In addition, children carrying 2 >500 kb copy number variations of unknown significance are 8 times more likely to have global developmental delay/intellectual disability than controls.^17^ The “two-hit” model mechanism has been proposed to explain the phenotypic variability associated with chromosome 16p12.1 microdeletions and distal 22q11.2 duplications.^25^ Girirajan et al^25^ observed that 14.8% of a cohort of 61 probands with 22q11.2 duplications had a second hit, a rate that is 38-fold higher than the general population. Li et al^28^ found additional copy number variations in 13% (2/15) of patients with a 22q11.2 deletion or duplication. Wincent et al identified an additional copy number variation in 31% of a cohort of 16 patients with distal 22q11.2 microduplication. From the cases described in Table 1, 30% (9/30) of affected individuals with distal 22q11.2 microduplications also carried a second copy number variant.

Our patient also had a second copy number variation in addition to the de novo 22q11.2 microduplication, which may have represented her “second hit”: This was a paternally inherited 0.25 Mb chromosome 4p14 duplication. This region contains the genes *FAM114A1, TMEM156*, and *KLHL5*, neither one of which is known to be disease causing nor preferentially expressed in the central nervous system. The chromosome 22q11.2 duplication in our patient includes approximately 35 genes. It is unknown whether one of these genes could be dosage sensitive and responsible for the observed phenotype. Five of these genes are identified in OMIM as disease causing: *UPB1* (β-ureidopropionase deficiency, OMIM 613161), *SPECC1 L* (facial clefting, oblique, 1, OMIM 600251), *MIF* (rheumatoid arthritis, systemic juvenile, susceptibility to, OMIM 604302), *IGLL1* (agammaglobulinemia 2, OMIM 613500), and *SMARCB1*. *SMARCB1* is of particular interest as it has been associated with autosomal dominant 15 mental retardation (OMIM 614608). SMARCB1 is a core component of an ATPase-dependent SWI/SNF (switch/sucrose nonfermenting) chromatin-remodeling complex (also known as the BAF complex), which plays an important role in several distinct processes such as transcription, cell differentiation, and DNA repair.^29^ Mutations in genes coding for key components of the SWI/SNF complex result in nonsyndromic and syndromic intellectual disability, including Coffin-Siris syndrome (OMIM 135900) and Nicolaides-Baraitser syndrome (OMIM 601358).^30,31^ Mutations in *SMARCB1* result in several forms of syndromic intellectual disability, including Coffin-Siris syndrome 3 and Kleefstra spectrum syndrome (OMIM 610253).^32^

This case illustrates the considerable challenges in interpreting copy number variations. Review of the phenotype reported in individuals carrying distal 22q11 microduplication involving similar LCR failed to establish any clear genotype–phenotype correlation. Direct pathogenicity of the microduplication is difficult to ascribe, given that the majority of parental carriers have a normal phenotype. However, the enrichment of this microduplication in patients with neurodevelopmental disabilities and the higher occurrence of a second copy number variation in affected carriers suggest that the distal 22q11 microduplications can act as a susceptibility/risk locus for neurodevelopmental disability. A “second hit” such as an additional genetic or environmental factor may be required to result in an abnormal neurodevelopmental phenotype.

## Supplementary Material

Supplementary material
